# Supplementary material for: From sequence to enzyme mechanism using multi-label machine learning
Source: BMC Bioinformatics. 2014 May 19;15:150. doi: 10.1186/1471-2105-15-150 (PMC4229970; doi:10.1186/1471-2105-15-150)
Supplement: Additional file 2 — Java code of ml2db. Additional file ml2db_code.tar.gz contains the Java source code to run the multi-label machine learning experiments and save the results to database. The code’s Javadoc is included. [file 1471-2105-15-150-S2.zip › additional file 2/ml2db/ml2db/doc/mulan/classifier/MultiLabelOutput.html]

MultiLabelOutput


JavaScript is disabled on your browser.


- Overview
- Package
- Class
- Use
- Tree
- Deprecated
- Index
- Help

- Prev Class
- Next Class

- Frames
- No Frames

- All Classes

- Summary:
- Nested |
- Field |
- Constr |
- Method

- Detail:
- Field |
- Constr |
- Method


mulan.classifier

## Class MultiLabelOutput

- java.lang.Object
- - mulan.classifier.MultiLabelOutput

- ---

    

  ```
  public class MultiLabelOutput
  extends java.lang.Object
  ```

  Class representing the output of a `MultiLabelLearner`.
  This can be a bipartition of labels into `true` and `false`,
  a ranking of labels, or an array of confidence values for each label.

  Author:
  :   Grigorios Tsoumakas

- - ### Constructor Summary

    Constructors

    Constructor and Description

    `MultiLabelOutput(boolean[] bipartition)`

    Creates a new instance of `MultiLabelOutput`.

    `MultiLabelOutput(boolean[] bipartition,
    double[] someConfidences)`

    Creates a new instance of `MultiLabelOutput`.

    `MultiLabelOutput(double[] probabilities)`

    Creates a new instance of `MultiLabelOutput`.

    `MultiLabelOutput(double[] probabilities,
    double threshold)`

    Creates a new instance of `MultiLabelOutput`.

    `MultiLabelOutput(int[] ranking)`

    Creates a new instance of `MultiLabelOutput`.
  - ### Method Summary

    Methods

    | Modifier and Type | Method and Description |
    | --- | --- |
    | `boolean` | `equals(java.lang.Object mlo)` Tests if two MultiLabelOutput objects are equal |
    | `boolean[]` | `getBipartition()` Gets bipartition of labels. |
    | `double[]` | `getConfidences()` Gets confidences of labels. |
    | `int[]` | `getRanking()` Gets ranking of labels. |
    | `boolean` | `hasBipartition()` Determines whether the `MultiLabelOutput` has bipartition of labels. |
    | `boolean` | `hasConfidences()` Determines whether the `MultiLabelOutput` has confidences of labels. |
    | `int` | `hashCode()` |
    | `boolean` | `hasRanking()` Determines whether the `MultiLabelOutput` has ranking of labels. |
    | `static int[]` | `ranksFromValues(double[] values)` Creates a ranking form specified values/confidences. |
    | `java.lang.String` | `toString()` |

    - ### Methods inherited from class java.lang.Object

      `getClass, notify, notifyAll, wait, wait, wait`

- - ### Constructor Detail


    - #### MultiLabelOutput

      ```
      public MultiLabelOutput(boolean[] bipartition)
      ```

      Creates a new instance of `MultiLabelOutput`.

      Parameters:
      :   `bipartition` - bipartition of labels

      Throws:
      :   `ArgumentNullException` - if bipartitions is null.


    - #### MultiLabelOutput

      ```
      public MultiLabelOutput(int[] ranking)
      ```

      Creates a new instance of `MultiLabelOutput`.

      Parameters:
      :   `ranking` - ranking of labels

      Throws:
      :   `ArgumentNullException` - if ranking is null


    - #### MultiLabelOutput

      ```
      public MultiLabelOutput(double[] probabilities,
                      double threshold)
      ```

      Creates a new instance of `MultiLabelOutput`. It creates a ranking
      based on the probabilities and a bipartition based on a threshold for the probabilities.

      Parameters:
      :   `probabilities` - score of each label
      :   `threshold` - threshold to output bipartition based on probabilities

      Throws:
      :   `ArgumentNullException` - if probabilities is null


    - #### MultiLabelOutput

      ```
      public MultiLabelOutput(double[] probabilities)
      ```

      Creates a new instance of `MultiLabelOutput`. It creates a ranking
      based on the probabilities.

      Parameters:
      :   `probabilities` - score of each label

      Throws:
      :   `ArgumentNullException` - if probabilities is null


    - #### MultiLabelOutput

      ```
      public MultiLabelOutput(boolean[] bipartition,
                      double[] someConfidences)
      ```

      Creates a new instance of `MultiLabelOutput`.

      Parameters:
      :   `bipartition` - bipartition of labels
      :   `someConfidences` - values of labels

      Throws:
      :   `ArgumentNullException` - if either of the input parameters is null or
          their dimensions do not match
  - ### Method Detail


    - #### getBipartition

      ```
      public boolean[] getBipartition()
      ```

      Gets bipartition of labels.

      Returns:
      :   the bipartition


    - #### hasBipartition

      ```
      public boolean hasBipartition()
      ```

      Determines whether the `MultiLabelOutput` has bipartition of labels.

      Returns:
      :   `true` if has bipartition; otherwise `false`


    - #### getRanking

      ```
      public int[] getRanking()
      ```

      Gets ranking of labels.

      Returns:
      :   the ranking


    - #### hasRanking

      ```
      public boolean hasRanking()
      ```

      Determines whether the `MultiLabelOutput` has ranking of labels.

      Returns:
      :   `true` if has ranking; otherwise `false`


    - #### getConfidences

      ```
      public double[] getConfidences()
      ```

      Gets confidences of labels.

      Returns:
      :   the confidences


    - #### hasConfidences

      ```
      public boolean hasConfidences()
      ```

      Determines whether the `MultiLabelOutput` has confidences of labels.

      Returns:
      :   `true` if has confidences; otherwise `false`


    - #### ranksFromValues

      ```
      public static int[] ranksFromValues(double[] values)
      ```

      Creates a ranking form specified values/confidences.

      Parameters:
      :   `values` - the values/confidences to be converted to ranking

      Returns:
      :   the ranking of given values/confidences


    - #### equals

      ```
      public boolean equals(java.lang.Object mlo)
      ```

      Tests if two MultiLabelOutput objects are equal

      **Overrides:**
      :   `equals` in class `java.lang.Object`

      Parameters:
      :   `mlo` - a MultiLabelOutput object

      Returns:
      :   true if the given object represents a MultiLabelOutput equivalent to this MultiLabelOutput, false otherwise


    - #### toString

      ```
      public java.lang.String toString()
      ```

      **Overrides:**
      :   `toString` in class `java.lang.Object`


    - #### hashCode

      ```
      public int hashCode()
      ```

      **Overrides:**
      :   `hashCode` in class `java.lang.Object`


- Overview
- Package
- Class
- Use
- Tree
- Deprecated
- Index
- Help

- Prev Class
- Next Class

- Frames
- No Frames

- All Classes

- Summary:
- Nested |
- Field |
- Constr |
- Method

- Detail:
- Field |
- Constr |
- Method
